# How Old Is My Dog? Identification of Rational Age Groupings in Pet Dogs Based Upon Normative Age-Linked Processes

**DOI:** 10.3389/fvets.2021.643085

**Published:** 2021-04-27

**Authors:** Naomi D. Harvey

**Affiliations:** Canine Behaviour and Research Department, Dogs Trust, London, United Kingdom

**Keywords:** dog, aging, behavior, development, age groups, geriatric, senior, puppy

## Abstract

Behavioral development is a lifelong process where cognitive traits such as learning and memory may be expected to take quadratic or linear trajectories. It is common practice for operational purposes to reduce study subjects into chronological categories when conducting research. However, there are no agreed-upon thresholds for this practice, and the lack of standardization may hinder comparison between studies of normative and pathological aging. In this perspective review, chronological categories have been identified that can be considered to represent normative cognitive and neurological aging in domestic family dogs. These categories work to capture age-related developmental trajectories for the majority of dog breeds. It is encouraged that researchers studying cognition and behavior, pathological cognitive deficits, or welfare of dogs across age categories utilize the categories presented here to best enable comparison between studies. The proposed groups could also support education programs informing owners of what behavioral changes to expect in their dog as they age, but they cannot be used to reflect health-based needs associated with breed-specific morbidity. The use of the age categories proposed here highlights significant welfare issues for breeds with the shortest average lifespans (e.g., the Great Dane). Studies show no evidence of an increased rate of behavioral or cognitive aging in short-lived breeds, and the shortest-lived breeds are most likely to die when classified by the proposed categories as Mature Adults. Adoption of these chronological categories in future research would aid comparison between studies and identification of non-normative age-related pathologies.

## Introduction

**Figure 1:**
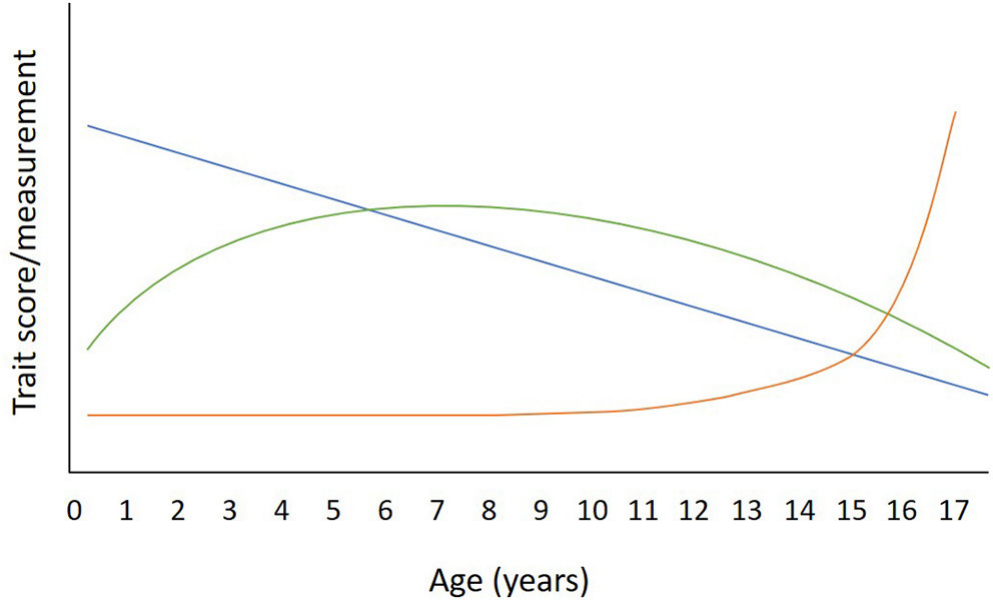
Illustrative example of three different types of relationships that behavioral traits could have with age in the dog. A linear relationship is illustrated in blue. An example of a trait that has a linear relationship with age would be activity/excitability, which peaks in puppyhood and declines steadily throughout the lifespan [e.g., (6)]. The Green arc illustrates a quadratic relationship, an example of which is attentiveness, which peaks in early adulthood and declines steadily thereafter [e.g., (7)]. The Orange line illustrates a trait that remains relatively stable until late life, when a steep change may be seen. An example of this trajectory is seen with the signs of canine cognitive dysfunction, which rises steadily from age 10 years, then rapidly after the age of 15 years (8).

Aging is a continuous, lifelong process that impacts health, behavior, and care requirements in humans and non-human animals. In the domestic dog, the diversity of physical conformation and breed-related features (such as body size) means that aging can impact dogs in varying ways, dramatically effecting longevity and morbidity (1–5). The impact of aging on behavior is also not straightforward (Figure 1), as age can have a linear relationship with certain behavioral traits [e.g., (6, 9)] but a quadratic relationship with others [e.g., (7, 10, 11)] or exhibit a steep change in later life [e.g., (6, 12)]. Because of the impact of age on behavior and health, age must always be considered in any study that includes dogs at differing stages of development. In practical terms, most research studies tackle this by grouping dogs into chronological age categories (often treating them as linear variables). However, as raised in a comprehensive review of studies related to dog aging, there is a distinct lack of standardization in research when grouping dogs by age, which hinders cross-study comparisons (13).

Across the mammalian kingdom, when comparing species, larger body size is well-established as a predictor of a longer average lifespan [e.g., (14)]. However, within species, individuals with larger absolute body mass tend to be shorter-lived [e.g., rodents (15) and humans (16)]. This is the case for the domestic dog, where larger breeds die at younger ages, due largely to an accelerated rate of growth between birth and adult size (17), which incurs growth-induced cellular damage *via* oxidative processes that reduce longevity (17–19). Large breeds can also be predisposed to a considerable number of inherited diseases related to their conformation, such as cardiovascular diseases, which contribute to their early mortality and morbidity (1) in addition to higher inbreeding coefficients, which are also associated with reduced longevity (5, 20).

Because large-breed dogs can be expected to develop health problems and die younger, the age dogs are considered “old” is often adjusted for the breed. For example, according to the 2019 American Animal Hospital Association's Canine Lifestage Guidelines, dogs can be considered senior during the last 25% of their estimated lifespan (21), which is heavily breed-dependent [e.g., (3)]. This means that veterinarians may consider large and giant breeds to require “geriatric care” when aged just 5 years old. However, categorizing shorter-lived breeds as geriatric when arguably still young is based upon physical health needs and may not reflect age-related processes, including behavioral or cognitive aging. Indeed, two studies that have evaluated breed differences in behavioral aging both found no evidence for an increased rate of change in the behavior of large, short-lived breeds (10, 22) with all dogs, irrespective of their anticipated lifespan, expected to follow the same lifespan trajectory. Using the term “geriatric” to describe dogs with shorter anticipated lifespans may serve to normalize their early mortality, thus masking breed-associated welfare issues.

In addition to the lack of standardization in age categories currently used, there are also issues of interpretability for utilizing existing categories. Often, the same threshold is shared by different categories, for example, the nomenclature 3–5 and 5–8 years, which makes it unclear which category a 5-year-old dog would belong to. Therefore, this perspective review aims to identify a standard of rational chronological categories based upon normative developmental and age-linked processes that could be applied for use in dog behavior research to better promote standardization and cross-study comparisons. The research was initially identified by searching for the terms “dog” and “age” in the title field on ScienceDirect.com. Papers were included here if they presented empirical data on cognitive or behavioral differences between groups or classifications of dogs based on age, in addition to data on age-related mortality. Additional research works were then searched for by checking paper titles in the reference lists of eligible manuscripts, as well as by searching for papers that had cited eligible manuscripts.

Although this review aims to identify rational chronological categories for use in behavioral research, it must be noted that such categories are not recommended for use in predicting health needs because the thresholds would need adjusting for differing life expectancies between populations due to the reasons discussed earlier. For health purposes, the “frailty index” for biological aging is becoming more common in human medicine, reflecting the severity of accumulated physical and cognitive illness that serves as good predictors of individual life expectancy [e.g., (23)]. Frailty indices provide a reliable method of predicting life expectancy, translating between populations where chronological age may not (24). A frailty index was recently developed for the dog and is more predictive of mortality than chronological age, acting as a better representation of health status (25). The use of the dog frailty index would better inform medical choices and interventions for individual dogs in practice than the use of age category and would allow dogs to be classified as frail when still considered mature adults, thus avoiding misuse of the term “geriatric” for what are objectively still young dogs.

### Early Life in the Dog

Early development in the dog can be split into five periods: prenatal (26), neonatal (birth to 12–13 days), transitional (14–20 days after birth), socialization (21–84 days), and juvenile (12 to 52–104 weeks after birth) (27). The juvenile period is the longest period of development, incorporating adolescence, which is when the infant transitions into the adult to become sexually and behaviorally mature [for an overview of the adolescent period of development in mammals, see (28)]. However, precisely when behavioral maturity is reached is a considerably under-researched question, as are the factors that influence maturation rate differences.

The mammalian brain undergoes considerable neurological reorganization during adolescence (29, 30). Neurological adolescent development may begin before puberty and can end a considerable time after it [e.g., in humans adolescence begins at around age 10 years and ends in our mid-20s (29)]. Puberty (the period of sexual maturation) in the dog is considered to occur between 6 and 9 months of age in males and 6–16 months in females (31, 32); however, behavioral and social maturity may be reached between 12 and 24 months of age and is suspected to differ depending on the breed (33, 34). There is also evidence to show that different behavioral traits exhibit different developmental and lifespan trajectories (12, 35). In terms of memory, dogs aged under 24 months have been shown to have the shortest memory span compared with older dogs (36), implying that memory is still undergoing a developmental change in this period.

For most lifespan studies of dogs, breaking down early development into the initial three post-birth periods (neonatal, transitional, and socialization) is not particularly useful unless the authors are specifically evaluating these periods. Puberty is a significant period of development for any animal and can be expected to impact dog behavior [e.g., (37)], so it would be rational to set the earliest age-group for a lifespan category as between birth and the expected onset of puberty: 0 to 6 months (26 weeks). Dogs in this age group could be classified as “Puppies,” with the majority realistically expected to be pre-pubertal.

The majority of dogs aged between 6 months to 1 year (27–52 weeks) could be presumed to be undergoing puberty (although it is acknowledged that data on the exact timing of puberty and breed-related differences are sparse). Thus, those aged between 6 months to 1 year (27–52 weeks) could be classified as “Juvenile,” capturing the period of change associated with the onset of puberty.

The limited data available on the timing of behavioral maturation in dogs suggest that adolescent development may continue until dogs are ~2 years of age, as they still exhibit behavioral changes between the age of 1 and 2 years (7, 36). Therefore, dogs that are 1 year old (aged between 12 and 24 months) could be classified as being “Young adults.” Previous research has used a similar structure, with dogs aged 6–12 months classified as being in “late puppyhood” and dogs aged 1–2 years as “adolescents” (7, 38).

An alternative broader grouping, if needed, would be to have two categories with puppies aged 0–25 weeks and adolescents aged 26 weeks to 2 years, as has been proposed by Wang and colleagues (39). However, a two-stage classification would not allow for evaluation of the impact of puberty on behavior in dogs, of which there is currently limited research.

### Middle and Late Life

Assuming a dog can be considered a “Mature adult” when they are 2 years of age, at what point can we consider them to be classed as senior or geriatric? Typically, the term geriatric is used to refer to individuals at the older end of the spectrum who are most likely to be experiencing health problems and cognitive impairments, whereas senior is used to describe old but relatively healthy individuals.

Seven years of age is commonly used as a threshold for the beginning of “old age” in dogs [e.g., (13, 40, 41)]. However, a number of more recent studies have used 8 years as a threshold for “senior” (e.g., 7, 8, 37)]. Although 8 years could be a good age to set this threshold, it is based upon classification of periods for one breed, the Border Collie, and the source material explaining the classification choice (42) is not in primary literature or accessible to this author. However, empirical support for setting 7 years as the beginning of old age comes from a recent study correlating age groups in dogs against signs of DNA aging (39), which led the authors to suggest that dogs between 2 and 6 years of age can be considered mature adults, and dogs aged 7+ years can be safely considered senior. The study did not account for extremely aged dogs by providing a geriatric group, however, and again, was based on just one breed.

One of the ways we can measure aging in the dog is to look at changes in learning and memory, as learning rate slows and becomes less flexible with increasing age (9). A cross-sectional study of visuospatial learning and memory impairments in beagles found that impairments were negligible in dogs aged 1 to 5 years but increased steadily from “middle age” (dogs aged 6 and 7 years) to 12 years (43); however, they did not test any dogs aged older than 12 years. These results could be considered to support the suggestion that dogs should be considered “Mature adults” when aged 2–6 years.

A study of cognitive abilities in beagles categorized dogs as “old” between 8 and 11 years and “senior” dogs as 11–14 years (44). They aimed to see how age impacted reversal learning ability to further understand age-related deficits in executive function. Although the “old” and “senior” dogs did not differ from each other in the number of errors or trials taken to reach the criterion, the “senior” dogs did represent a distinct group of dogs when considering the types of errors made. Dogs in the “old” group made errors indicative of an impaired ability to learn new stimulus–reward rules. Dogs in the “senior” group also made errors in stimulus–reward learning but made additional errors indicative of perseverative behavior, where they were less able to inhibit their original responses to the previously rewarded stimulus (44). These behaviors are thought to be controlled in the prefrontal cortex, which suggests this area of the brain may be vulnerable to aging (44). In support of this theory, the same authors reported in a separate study that frontal lobe size is significantly reduced in old and senior dogs compared with that in younger adults (45).

In a clinical study of cognitive deficits and neurological abnormalities in aging dogs (40), dogs with neurological abnormalities were shown to have a median age of 13 years, and dogs with diagnosed canine cognitive dysfunction syndrome [an age-related pathology of compromised behavior (13)] had a median age of 12 years. A cross-sectional study of behavior in normally aging dogs (excluding those with known or suspected canine cognitive dysfunction or neurological abnormalities) identified marked changes in multiple behavior for dogs aged >12 years compared with dogs aged 10–12 or <10 years (46). An interview study of owners of dogs aged older than 9 years classified dogs into three unlabeled age groups (9–11, 12–14, and 15–17 years) and found that each group showed a greater incidence of cognitive impairment than the previous (47). However, there were no dogs with “severe” cognitive impairments in the 9–11 age group compared with 3.3% aged 12–14 years classified as “severe” and 14.3% of the 15–17-year-old population.

Looking at mortality, in a study of owned dogs in England that died between 2009 and 2011 (3), the median longevity was 12 years, with an interquartile range of 9 to 14 years, meaning that half of United Kingdom pet dogs were still alive when aged 12 years. However, for certain breeds, the median longevity values were as low as 5.5 (Dogue de Bordeaux) and 6 years (Great Dane). A total of 22/36 breeds had a median longevity of 12+ years, representing 68.8% of all individual dogs in the studied population. An earlier study, published in 1999, of longevity in United Kingdom dogs reported the mean age at death for natural causes at 12 years of age, with only 8% of dogs living beyond the age of 15 years (48). In contrast to these findings, a more recent study, published in 2020, of owned dogs in the United States revealed higher median longevity of 15.4 years (5). Such differences may be due to differences in breed composition among the populations studied, genetic differences between geographically isolated populations within breeds, or advances in veterinary medicine.

In accepting that dogs should be considered “Mature adults” when aged between 2 and 6 years, it is suggested here that dogs aged 7–11 years could be considered “Senior,” whereas dogs aged 12+ years be considered “Geriatric” based upon the greater incidence of cognitive impairments in dogs of that age (13, 40, 46, 47) and the greater likelihood of death past the age of 12 years (3, 48). For studies of senior dogs where finer detail is desired, the senior period between 7 and 11 years of age could be split into Early-senior (7–9 years) and Late-senior (10–11 years), as significant differences have been found between dogs in these age groups (7, 43, 46).

A final category could be added for dogs aged 15+ years to be considered “Very aged.” Due to survivor bias and ethical issues associated with studying dogs in this age group, it is rare to see dogs older than 15 years as participants in behavior or cognition studies, hence why the category of 12+ years as “geriatric” may be more appropriate for most purposes. The exception may be where data are collected from existing records (such as veterinary records) or by owner questionnaire or interview. Pathological cognitive declines become markedly more common in this age category. Neilson and colleagues evaluated signs of cognitive impairment in dogs aged 11–12, 13–14, and 15–16 years and found that dogs aged 15 and 16 years had a much greater incidence of cognitive impairment compared with both other age groups (49). In a group of Japanese dogs of various breeds, the physical signs of canine cognitive dysfunction, a behavioral syndrome affecting old dogs, increased steadily from the age of 10 years, whereas confirmed diagnoses of canine cognitive dysfunction increase sharply from 15 years of age (8). Severe cognitive impairments have also been found more commonly in dogs aged 15–17 years, with 47.6% of dogs in this age group showing some signs of cognitive impairment (47).

## Discussion

**Figure 2:**
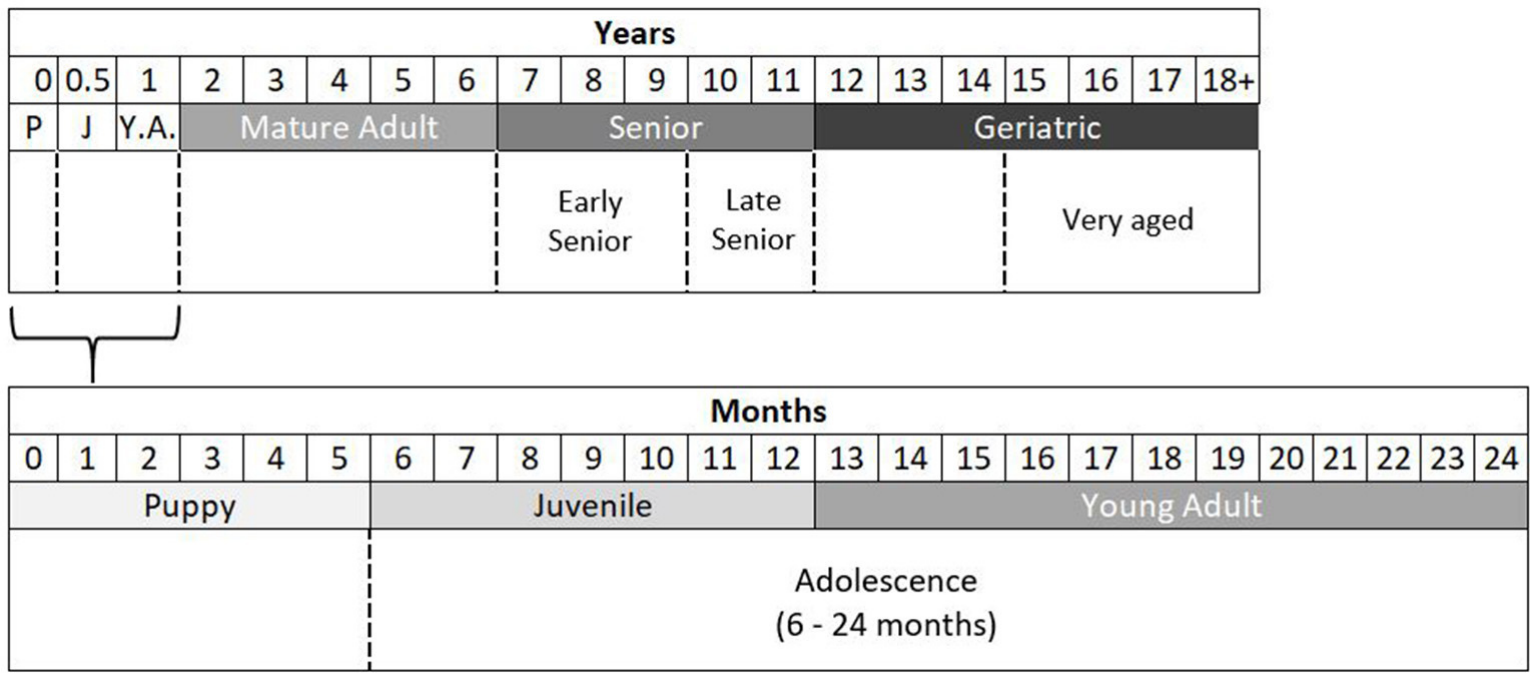
Normative and developmental stage thresholds for categorizing the domestic dog into age groups. Shown in dashed lines on the bottom rows are optional broader/finer level groupings. P, puppy; J, juvenile; Y.A, young adult.

Based upon the literature reviewed here, which the author acknowledges is not exhaustive, the following flexible category system is presented for use in research as a rational way to group dogs according to age, based upon normative aging and developmental stages (Figure 2). A basic six-category system is suggested, with optional broader or finer groupings that can be used depending upon the research's nature. Under this system, Puppies are aged 0–6 months (0–26 weeks), Juveniles are dogs in the pubertal period, aged 6 months to 1 year (27–52 weeks), and Young Adults aged 1 year (12–24 months) are fully grown, typically post-pubertal dogs that are still undergoing adolescent development. An option here would be to categorize dogs aged 6–24 months as Adolescent if sample size is a limitation. Dogs aged 2–6 years can be considered Mature Adults, and those aged 7–11 years Senior. A finer detail option would be to categorize dogs aged 7–9 years as Early-Senior and dogs aged 10–11 years as Late-Senior. For most purposes, dogs aged 12+ years can be considered Geriatric, but this period can be enhanced with a subclassification of Very-Aged dogs that are 15 years or older.

Certain breeds will likely have no or few representatives in the Geriatric or Very-Aged groups proposed here (e.g., Great Dane and Dogue de Bordeaux). Although short-lived breeds have higher early mortality rates, evidence suggests that they are not behaviorally and neurologically geriatric when they die (10, 22). Classification systems where the threshold for senior and geriatric are adjusted according to the expected lifespan of the breed may certainly be useful for predicting health problems but may also help to “normalize” what should be considered pathological aging and health problems related to the inherited disease within breeds with early mortality. Should the classifications suggested here be used to describe all dogs, it becomes clear that dogs with early mortality are largely dying when Mature Adults, or at best, early into the Senior period, and they should be described as such so as not to mask the severity of the problems associated with early mortality in short-lived breeds. Morbidity and mortality when old are acceptable, indeed, inevitable. However, whenever we see high morbidity and mortality in an animal that can be objectively classified as young, it should concern us. Although short-lived breeds may be aging typically for their breed, they are aging atypically for their species. By classifying short-lived breeds as dying when “geriatric,” we give the situation a free pass, allowing them to be thought of as old. Unfortunately, the fact of the situation is that such breeds are dying earlier than they should be for their species, due to inherited breed-based defects.

The research drawn on in this work was largely cross-sectional, which means that age may be confounded by cohort effects, which is not ideal for the study of age-related processes. More robust insights into age-associated processes in dogs will come from longitudinal cohort studies, such as the Dogslife project (50), Generation Pup (51), and the Golden Retriever Lifetime Study (52).

Although this study aimed to define rational age categories for operational research and classification purposes, it must be remembered that age is a continuous variable. Some behavioral and cognitive traits may be expected to decline linearly with increasing age from adulthood (6, 9, 11) or to develop quadratically through the lifespan of a dog (6, 7, 10, 11). The categories proposed here could be of use in behavioral and cognitive research, veterinary behavioral medicine, or to support education programs informing owners of what behavioral changes to expect in their dog as they age. The use of these categories in population-based research where the aim is to compare between age groups could greatly enhance the ability to draw comparisons between studies to enable greater insights into aging in the dog.

## Data Availability Statement

The original contributions presented in the study are included in the article/supplementary material, further inquiries can be directed to the corresponding author/s.

## Author Contributions

NH conceived, wrote, and revised this manuscript.

## Conflict of Interest

The author declares that the research was conducted in the absence of any commercial or financial relationships that could be construed as a potential conflict of interest.
